# Meat Intake and Insulin Resistance in Women without Type 2 Diabetes

**DOI:** 10.1155/2015/174742

**Published:** 2015-07-09

**Authors:** Larry A. Tucker, James D. LeCheminant, Bruce W. Bailey

**Affiliations:** ^1^Department of Exercise Sciences, Brigham Young University, 237 SFH, Provo, UT 84602, USA; ^2^Department of Exercise Sciences, Brigham Young University, 269 SFH, Provo, UT 84602, USA; ^3^Department of Exercise Sciences, Brigham Young University, 267 SFH, Provo, UT 84602, USA

## Abstract

*Purpose*. To examine the relationship between meat intake and insulin resistance (IR) in 292 nondiabetic women. *Methods*. IR was evaluated using the homeostasis model assessment (HOMA). Diet was assessed via 7-day weighed food records. Servings of very lean meat (VLM) and regular meat (meat) were indexed using the ADA Exchange Lists Program. Physical activity was assessed using accelerometers and body fat was measured using the Bod Pod. *Results*. Meat intake was directly related to HOMA (*F* = 7.4; *P* = 0.007). Women with moderate or high meat intakes had significantly higher HOMA levels than their counterparts. Adjusting for body fat weakened the relationship (*F* = 1.0; *P* = 0.3201). Odds ratio results showed that the low meat quartile had 67% lower odds of being IR (75th percentile) compared to their counterparts (OR = 0.33; 95% CI = 0.16–0.71). These findings changed little after adjusting for all covariates simultaneously (OR = 0.34; 95% CI = 0.14–0.83). Conversely, VLM intake was not related to HOMA, with or without the covariates. *Conclusion*. Moderate and high meat intakes are associated with increased insulin resistance in nondiabetic women. However, differences in body fat contribute significantly to the relationship. VLM is not predictive of IR. Prudence in the amount and type of meat consumed may be helpful in decreasing the likelihood of IR.

## 1. Introduction

Type 2 diabetes is a significant risk factor for numerous diseases and its prevalence in adults has risen in recent years [1]. Insulin resistance and the resultant increase in insulin output by pancreatic beta cells are associated with several metabolic abnormalities and almost always precede the development of type 2 diabetes [2]. Further, insulin resistance is associated with the metabolic syndrome and independently increases the risk of CVD [3–6]. Hence, reducing the incidence of insulin resistance has significant potential health implications.

Scientific evidence suggests a link between dietary habits and both the incidence of type 2 diabetes [7–10] and insulin resistance [7, 11, 12]. In general, a “western” dietary pattern that includes red and processed meats, foods, and beverages with high sugar content, refined grains, high fat and fried foods, and/or excessive snacking appears to elevate the risk of type 2 diabetes [8, 10] as well as insulin resistance [7, 11, 12].

Emerging evidence suggests that meat intake alone may be a significant predictor of type 2 diabetes [10, 13]. For example, a recent meta-analysis of 12 cohort studies by Aune et al. indicated 17%, 21%, and 41% increases in risk of type 2 diabetes when comparing high versus low intake of total meat, red meat, and processed meats, respectively [13]. Recent work also suggests that high consumption of some types of meat increases the risk of developing the metabolic syndrome [6].

Per capita meat consumption has increased in the U.S. and worldwide, with red meat accounting for the largest portion of total meat intake [14]. Further, the prevalence of hyperinsulinemia in nondiabetic US adults has increased [15]. These changes likely point to higher levels of insulin resistance in adults without diabetes and greater risk for CVD.

To prevent type 2 diabetes, the best strategy is probably to minimize the development of insulin resistance. Unfortunately, little work has been performed investigating the contribution of meat intake on the development of insulin resistance. Moreover, to date, methodological shortcomings have surfaced in many studies conducted to examine the meat and insulin resistance connection. Specifically, few, if any, studies have controlled for differences in body fat. Yet body fat plays a significant role in the development of insulin resistance [4]. Furthermore, to date, investigations have failed to control for differences in physical activity, particularly objectively measured activity. Yet lack of physical activity is an important factor in the development of insulin resistance [16]. Lastly, the vast majority of studies have measured diet using the 24-hour recall or food frequency questionnaires [13]. Yet, participants often have difficulty recalling precisely what they have eaten in the past, and additional biases are introduced when participants are required to estimate serving sizes.

The present investigation was designed to minimize these methodological weaknesses. A recent review by Okorodudu et al. suggested that BMI lacks sensitivity to identify excessive adiposity [17]. Rather than using body weight or BMI, air displacement plethysmography (Bod Pod) was employed to assess body fat. Additionally, physical activity was measured objectively using accelerometers, rather than ignoring differences in physical activity or using self-reported estimates. Furthermore, energy intake and dietary intake were assessed using 7-day weighed diet records. Given these high quality measurement methods, the primary objective was to determine the extent to which meat intake is associated with insulin resistance in middle-aged women.

## 2. Materials and Methods

### 2.1. Participants

This study utilized a cross-sectional design. Each participant provided consent to be a research participant using an informed consent document approved by the Institutional Review Board of the university. Participants for this study included a sample of 292 women recruited from approximately 20 cities in two metropolitan areas of the Mountain West, using fliers, newspaper advertisements, and word of mouth. Interested participants were included in the study if they were 35–49 years of age and physically healthy, determined by a physical activity readiness questionnaire (PAR-Q). Participants were excluded if they were diabetic, smoked or used tobacco products, were pregnant, or were lactating.

### 2.2. Procedures

Each participant reported to the university Human Performance Laboratory for testing on two separate occasions separated by at least one week. During the first testing session, the informed consent document was signed, the study protocol was explained, questions were answered, and each participant was measured for height, weight, and body fat percentage using the methods described below. After completion of the body composition assessment, each participant was fitted with an Actigraph accelerometer (Health One Technology, Inc., Fort Walton Beach, FL) and instructed to wear it continuously for the next seven days. Also during these same seven days, each participant was issued an Ohaus 2000 electronic scale (Florham Park, NJ) and 7 blank food records and asked to record all food and beverages consumed for the next week. Each participant was trained regarding how to weigh and record food and beverage intake using plastic food models. Lastly, each participant was given a blood requisition form and driving directions and instructed to visit the hospital laboratory in the next week for a blood draw, following a fast of 12 hours (water was allowed). A member of the research team contacted each participant during the week via telephone to ensure that the proper protocol was being followed.

### 2.3. Body Weight, Height, and Body Fat Percentage

Body weight and height were assessed using a digital scale (Tanita, Tokyo, Japan) and wall-mounted stadiometer (Seca, Chino, CA), respectively, in order to calculate BMI (kg/m^2^). Weight was assessed with participants in a standard, university-issued one-piece bathing suit. The Bod Pod (Life Measurement Instruments, Concord, CA), which utilizes air displacement plethysmography, was used to determine body fat percentage. The Bod Pod has been shown to be both valid and reliable for body composition determination [16, 18–20].

### 2.4. Insulin, Glucose, and HOMA-IR

To determine insulin resistance, the homeostasis model assessment of insulin resistance (HOMA) was utilized. Upon arrival at the hospital laboratory following a 12-hour fast, blood was taken from the antecubital vein of each participant, centrifuged at 4°C for 15 min at 2000 g, and stored in aliquots at −20°C. Fasting insulin levels (*μ*U/L) were determined using the Access Ultrasensitive Insulin assay (Beckman Coulter, Inc., Brea, CA). Fasting glucose levels were determined using the Dimension Vista System and the Flex reagent cartridge (Siemens, Deerfield, IL). Using these fasting insulin and glucose levels, HOMA was computed as follows: fasting insulin (*μ*U/mL) × fasting glucose (mg/dL)/405. HOMA is frequently used as a valid measure of insulin resistance as it has been shown to be comparable to the euglycemic clamp method [21]. More than 500 studies using HOMA have been published [22].

### 2.5. Dietary Intake

To determine food consumption, each participant weighed and recorded all food and beverages for seven consecutive days. This coincided with the same 7 days in which physical activity was monitored. All food and beverages were weighed using an Ohaus 2000 electronic scale (Florham Park, NJ). Participants were asked to continue with their normal dietary patterns during the recording period.

All dietary intake assessment measures have strengths and weaknesses [23]. Seven-day weighed food records have the following distinct strengths: (1) high precision, (2) good index of foods typically consumed, and (3) eliminating the need for memory, by recording food as it is consumed and minimizing error associated with estimating serving sizes, which tends to reduce over- and underreporting [23, 24]. Commonly cited weaknesses of weighed food records include reactivity and high participant burden.

After completion of the 7-day weighed food records, a member of the research team reviewed with the participant the accuracy of each food item listed for each day. Subsequently, all energy, macronutrient, and food intakes were analyzed using the ESHA software (Salem, OR). The ESHA software is often utilized in research studies as a valid program for analysis of energy and macronutrient intake [25]. After analysis, if a participant's food intake was less than 130% of estimated resting metabolic rate, as determined using the Ravussin formula based upon lean body mass [26], she was asked to redo her food intake for the week.

*Meat Intake*. This study focused on meat intake. Servings were defined according to the American Dietetic Association and the American Diabetes Association (ADA) Exchange Lists Program. The American Dietetic Association is now called the Academy of Nutrition and Dietetics. ESHA software was used to analyze each meat exchange based on the fat content and grams of protein per serving. According to the 2003–2007 ADA Exchange Lists Program, one meat exchange is equivalent to 1 ounce of meat, no carbohydrate, and 7 g of protein. However, the fat content in g is used to distinguish the type of meat exchange, including very lean (0-1 g fat), lean (3 g fat), medium fat (5 g fat), and high fat (8 g fat) meats. Examples of meat in the “very lean” (VLM) category included skinless chicken, turkey, fish, and game. Meats in the remaining categories (not very lean meats) included mostly red and processed meats (meats).

Some of the meats included in the “very lean” category (VLM) have previously been reported to decrease the risk of type 2 diabetes [11], whereas servings in the meat category (red and processed meats) have been previously described as culprits in the risk of type 2 diabetes. Hence, analysis of meat consumption was separated into two categories, VLM and meat (non-VLM meat). If a meat serving contained 3 or more grams of fat, the extra fat grams were counted as part of the ADA fat exchange category. In addition, meat intake was reported per 4186 kJ (1000 kcal) to adjust for total energy intake and its potential influence on insulin resistance.

### 2.6. Physical Activity

Physical activity was assessed using the Actigraph accelerometer. Each participant wore an accelerometer for 7 consecutive days, attached at the left hip using a small pouch and belt. Participants wore the accelerometer during all waking and sleeping hours, except when in the water (i.e., bathing or swimming). Participants were asked to continue with their normal activity patterns. Physical activity was partitioned into 10-minute epochs or bouts based on recommendations from the American College of Sports Medicine and American Heart Association [27]. There were 144 10-minute epochs per day; thus, total physical activity was the sum of the 144 epochs over 7 days (1008 epochs) [28, 29]. Accelerometers have been reported to be valid, reliable, and objective measures of total physical activity [30, 31].

### 2.7. Data Analysis

Means and standard deviations were used to describe the key variables of the study. HOMA was used to index levels of insulin resistance. Key demographic, dietary intake, anthropometric, and other variables are shown in Table 1. To differentiate between levels of meat intake, meat servings were divided into quartiles and the two middle categories were collapsed forming three meat intake groups (low, moderate, and high) (Table 2). Similarly, VLM servings were divided into quartiles with the two middle quartiles combined also forming low, moderate, and high VLM intake groups (Table 3). Mean insulin resistance levels were compared across the three meat and VLM groups, with and without control of the potentially confounding variables. Means were adjusted for differences in the potential confounding variables and compared across groups using partial correlation and least squared means. Because HOMA data were not normally distributed, they were log-transformed. To facilitate interpretation of the results, HOMA data in Results and tables were reported in common clinical units, and the statistics and *P* values associated with the results of the log-transformed findings were reported with the associated clinical units.

As has been utilized elsewhere [11], insulin resistance was defined as HOMA levels ≥ the 75th percentile. Odds ratios were calculated using prevalence data to compare the likelihood of women with low meat intake (1st quartile) or low VLM intake (1st quartile) having insulin resistance compared to the other levels of meat or VLM consumption (moderate or high), respectively. Statistical significance was determined using 95% confidence intervals. The statistical comparisons, including the odds ratio results, were adjusted for differences in the potentially confounding variables, including age, education, percent of energy derived from carbohydrate, protein, and dietary fat, total energy intake, ADA exchanges for starch, sweets, fruit, nonstarchy vegetables, dairy, very lean meats, and dietary fat, BMI, body fat percentage, and objectively measured physical activity, considered individually and in combination, using the SAS Proc Logistic procedure (Table 4). Statistical significance was set at the *P* < 0.05 level.

## 3. Results

On average, participants in the present study were 40.3 ± 3.1 years old, normal weight (23.8 ± 3.3 kg/m^2^), with 31.6 ± 6.9% body fat. A total of 96% of the participants were Caucasian. Participants consumed a relatively typical American diet for percentage of energy from carbohydrate (55.4%), fat (30.5%), and protein (14.1%). Other descriptive variables (mean and standard deviation), along with the median, minimum, and maximum values, are presented in Table 1.

When comparing mean insulin resistance levels across meat intake groups, women in the high and moderate meat intake groups had significantly higher HOMA scores than those in the low meat intake group (*F* = 7.4; *P* = 0.0070). When age, education, carbohydrate intake, protein consumption, fat intake, total energy consumption, and exchanges of VLM, starch, sweets, fruit, nonstarchy vegetables, dairy, dietary fat, and physical activity were individually used as control variables, women in the high and moderate meat exchange categories continued to have significantly higher insulin resistance scores than participants in the low meat intake quartile (*P* < 0.05). However, when body fat percentage or BMI were controlled individually, differences in insulin resistance disappeared (*P* > 0.05). As shown in Table 2, when all of the potential confounding control variables were added to the model as statistical controls, mean differences in insulin resistance were not statistically significant (*F* = 1.9; *P* = 0.1672). When all covariates except body fat percentage were controlled statistically, mean differences in insulin resistance among the meat intake quartiles were significant (*F* = 6.2; *P* = 0.0136).

When comparing insulin resistance levels across the VLM groups, there was not a significant difference in HOMA (*F* = 1.0; *P* = 0.328). Adjusting for differences in the potential confounding variables, considered individually and in combination, did not influence the relationship between VLM intake and insulin resistance.

Two analysis strategies were used to evaluate the meat and IR relationship. Table 4 shows the odds of being in the insulin resistance group (HOMA ≥ 75th percentile) in women with low meat consumption (≤ 25th percentile) or low VLM consumption (≤ 25th percentile) compared to the other levels of meat or VLM consumption, respectively. Without statistical controls, participants who consumed the lowest amount of meat had 0.33 (95th CI = 0.16–0.71) times the odds of being insulin resistant compared to women with moderate or high levels of meat intake. Individually controlling for age, education, carbohydrate intake, protein consumption, fat intake, energy intake, physical activity, or any of the dietary exchange variables did little to alter the meat intake odds ratios. Adjusting for differences in BMI and body fat percentage individually weakened the odds ratios to the point of nonsignificance (*P* > 0.05). However, when all of the potential confounders were controlled simultaneously, the results were statistically significant. Specifically, women with low meat consumption had 0.34 (95% CI = 0.14–0.83) times the odds of being insulin resistant compared to the other women.

Also displayed in Table 4 are the results associated with consumption of VLM. Without statistical controls, participants who consumed low levels of VLM (1st quartile) did not have more or less odds of insulin resistance compared to their counterparts (OR = 1.15; 95% CI = 0.63–2.10). Controlling statistically for differences in age, education, carbohydrate intake, protein consumption, fat intake, energy intake, physical activity, or any of the dietary exchange variables did little to alter the VLM and HOMA odds ratios.

## 4. Discussion

The present study is one of only a few investigations to examine the connection between meat consumption and insulin resistance. High quality measurement methods were employed and the results showed that high and moderate meat consumption are significant predictors of insulin resistance in women without type 2 diabetes. From the covariate analyses, however, it appears that at least part of the relationship between meat intake and insulin resistance is a function of body fat. Conversely, after adjusting for differences in all of the potential confounding factors simultaneously, including BMI and body fat percentage, the odds of insulin resistance in those with low meat intake was only 1/3 of those in the moderate and high meat intake groups. Consumption of very lean meat (VLM) does not appear to be related to insulin resistance.

Meat is a broad food category that is easily identified and widely consumed by adults and, therefore, is meaningful for investigation. Nevertheless, an individual's total diet includes a mix of foods and macro- and micronutrients. Often as one food group or macronutrient increases or decreases, another changes proportionally in the opposite direction. Because of the interactivity of foods and nutrients, it is difficult to study the effect of specific foods or food groups on a health outcome in isolation.

To manage this issue, several potential confounding variables were controlled statistically in the present study. None of the potential confounders influenced the relationship between meat consumption and insulin resistance, except body mass index and body fat percentage. In short, independent of differences in age, education, carbohydrate intake, protein consumption, dietary fat intake, total energy consumption, servings of starch, sweets, fruit, vegetables, dairy, VLM, fat exchanges, and objectively measured physical activity, as meat consumption increased, insulin resistance tended to increase in middle-aged women, and as meat intake decreased, insulin resistance tended to decrease. Only BMI and body fat percentage, considered individually, influenced the meat and HOMA association meaningfully. Hence, it appears that the association between meat consumption, particularly red meat, and insulin resistance is driven, at least partly, by differences in body fat percentage in middle-aged women.

The findings of the present investigation are consistent with the results of a 2009 meta-analysis conducted by Aune et al. [13]. The review included 12 large investigations, 10 of which examined the association between red meat intake and type 2 diabetes. With combined results, the 10 studies showed that those with high red meat intake had 21% greater risk of developing type 2 diabetes than those with low intake (95% CI: 1.07–1.38). Overall, the literature appears to implicate red and processed meats as carrying more risk for type 2 diabetes than other types of meat. The present study also revealed differences in the relationship between type of meat consumed and insulin resistance. While VLM (i.e., chicken, fish, turkey, etc.) was not predictive of insulin resistance, consumption of other meats (i.e., red and processed meats) was related significantly to HOMA.

Our finding that meat intake is predictive of insulin resistance is also consistent with the significantly lower risk and prevalence of type 2 diabetes in vegetarians compared to omnivores [32–34]. It can be argued that vegetarians differ from meat eaters on factors other than meat consumption, including body weight and body fat. However, most studies investigating this link have controlled for differences in BMI, and the relationship has remained significant [13, 32–34].

A number of mechanisms could explain the relationship between meat intake and insulin resistance. For example, in previous research, meat consumption has been connected to heme-iron found in red meat. Iron tends to promote oxidative stress and may increase risk of type 2 diabetes [6, 35, 36]. Moreover, many processed meats contain nitrates and nitrites, which can be converted to nitrosamines, which have been found in animal investigations to increase the risk of diabetes [37, 38]. Additionally, high consumption of animal protein has been associated with increased risk of type 2 diabetes [39]. It appears that high levels of amino acids, which are abundant in red meats, interfere with normal metabolism of glucose, which can promote insulin resistance [40, 41].

Peppa et al. [42] indicate that glycotoxins may be a missing link that explains the relationship between dietary fat and meat intake in relation to risk of type 2 diabetes. Additionally, several studies [43, 44] point to the role of red meat and saturated fat intake and their contribution to inflammation, increasing risk of type 2 diabetes. Lastly, a number of investigations show that red and processed meats and dietary fat intake promote weight gain and obesity [45–48], which is a key risk factor in the development of insulin resistance and type 2 diabetes. Given the results of the present study, the pattern of high (red) meat intake, increased risk of obesity, followed by increased odds of insulin resistance and type 2 diabetes must be given special consideration.

Regarding mechanisms, the mediating effect of body fat percentage and BMI cannot be overlooked in the relationship between meat intake and insulin resistance of the present study. Obesity and body fat undoubtedly play a significant role in the development of both insulin resistance and type 2 diabetes [49]. As shown in the Aune et al. review [13], most studies examining meat intake and type 2 diabetes have adjusted for the influence of BMI, but very few have controlled for adiposity. Because the present study also adjusted for differences in body fat percentage, a side-by-side comparison of the mediating effects of body fat and BMI can be seen. From the results, it appears that body fat influences the meat and IR results to a greater extent than BMI.

Although body fat percentage seems to influence the meat and IR relationship more than BMI, BMI is known to account for a significant level of the variance in glucose disposal [49]. Also, it has previously been reported that insulin-mediated glucose uptake is significantly related to body fat percentage (*r* = −0.33 to −0.71) [4]. Findings from the present investigation indicate that both meat intake and body composition are important when considering risk for insulin resistance.

Physical activity has been shown previously to be a predictor of insulin resistance [50–52]. In the present study, adjusting statistically for differences in total physical activity was not sufficient to modify the relationship between meat and insulin resistance. It appears, therefore, that the meat and insulin resistance relationship is independent of physical activity.

The present study had several strengths, including a large sample size, control of several potential confounding variables, and the use of high quality, objective measurement methods. However, there were also limitations, including a cross-sectional study design, which prevents cause-and-effect conclusions. Furthermore, it should be noted that the present sample was comprised of women without type 2 diabetes and nonsmokers. Hence, it is possible that the relationship between meat intake and insulin resistance could be different in a less healthy sample. Additionally, HOMA is not the gold standard, but a surrogate method for indexing insulin resistance. Lastly, participants were predominately Caucasian women, all middle-aged. Hence, generalization of the results should probably be limited to groups with similar characteristics.

## 5. Conclusion

In conclusion, it appears that meat intake, particularly red and processed meats, is associated with higher levels of insulin resistance in middle-aged women without type 2 diabetes. Among the many potential confounders examined in this study, BMI and body fat percentage influenced the association significantly. Consequently, both a lower meat intake and lower levels of body fat appear important in reducing the likelihood of insulin resistance, especially in this sample. Consumption of very lean meats (VLM) does not seem to play a role in insulin resistance. To decrease the likelihood of insulin resistance, prudence in the amount and type of meat consumed may be helpful.

## Figures and Tables

**Table 1:** Descriptive information about all participants [*n* = 292].

Variable	Mean	SD	Minimum	50th percentile	Maximum
Age [years]	40.3	3.1	35.0	40.5	48.0
Fasting glucose [mg/dL]	86.9	7.2	73.0	87.0	111.0
Fasting insulin [*μ*U/mL]	7.4	4.4	1.3	6.5	34.8
HOMA	1.6	1.0	0.2	1.4	8.9
Carbohydrate [% total kJ]	55.4	6.4	25.4	55.9	73.3
Protein [% total kJ]	14.1	3.0	8.5	13.6	32.3
Fat [% total kJ]	30.5	5.8	11.6	30.5	51.6
Energy intake [kJ]	8510.0	1356.0	4771.9	8297.7	14623.5
Starch exchanges [servings]	3.7	0.9	0.4	3.6	7.1
Sweets exchanges [servings]	3.2	1.2	0.2	3.1	8.6
Fruit exchanges [servings]	1.2	0.8	0.0	1.0	4.1
Vegetables exchanges [servings]	0.7	0.5	0.0	0.6	2.9
Dairy exchanges [servings]	0.5	0.5	0.0	0.5	3.1
Meat exchanges [servings]	1.3	0.6	0.3	1.3	4.6
VLM exchanges [servings]	0.8	0.8	0.0	0.6	6.7
Fat exchanges [servings]	4.9	1.3	1.0	4.9	8.8
BMI [kg/m^2^]	23.8	3.3	15.8	23.7	32.1
Body fat [%]	31.6	6.9	14.6	32.0	44.8
Physical activity [counts]^*∗*^	268.8	79.8	82.8	265.0	494.6

*Note.* Each of the exchange variables was calculated as servings per 4186 kJ [1000 kcal].

VLM exchanges: servings of very lean meat. According to the exchange program, 1 exchange = 28 grams [1 oz].

^*∗*^Physical activity counts were divided by 1000 to make values more manageable. A mean of 268.8 reflects 2.688 million counts for the week of activity monitoring.

**Table 2:** Mean level of insulin resistance [HOMA] by meat exchange category, without and with means adjusted for potential confounders.

Variable controlled	Meat Exchange Group [*n* = 292]						*F*	*P*
	Low meat intake		Moderate meat intake		High meat intake			
	[1st quartile]		[2nd-3rd quartiles]		[4th quartile]			
	[*n* = 73]		[*n* = 146]		[*n* = 73]			
	Mean	SD	Mean	SD	Mean	SD		
None	1.39^a^	0.88	1.69^b^	1.02	1.71^b^	1.21	7.4	0.0070
Age	1.39^a^		1.69^b^		1.71^b^		7.3	0.0072
Education	1.40^a^		1.70^b^		1.73^b^		7.3	0.0075
Carbohydrate intake	1.41^a^		1.69^b^		1.68^a,b^		4.3	0.0389
Protein intake	1.39^a^		1.70^b^		1.68^a,b^		6.5	0.0113
Fat intake	1.39^a^		1.69^b^		1.70^a,b^		5.5	0.0197
Total energy intake	1.40^a^		1.68^b^		1.71^b^		7.1	0.0083
Starch exchanges	1.36^a^		1.70^b^		1.72^b^		8.8	0.0033
Sweets exchanges	1.38^a^		1.70^b^		1.69^b^		7.5	0.0067
Fruit exchanges	1.41^a^		1.69^b^		1.69^a,b^		5.5	0.0202
Vegetable exchanges	1.39^a^		1.69^b^		1.71^b^		7.3	0.0074
Dairy exchanges	1.37^a^		1.68^b^		1.74^b^		8.6	0.0036
VLM exchanges	1.39^a^		1.69^b^		1.71^b^		7.3	0.0071
Fat exchanges	1.38^a^		1.69^b^		1.71^b^		6.6	0.0107
BMI	1.51		1.66		1.65		1.8	0.1846
Body fat percentage	1.53		1.67		1.61		1.0	0.3201
Physical activity	1.39^a^		1.70^b^		1.69^b^		7.1	0.0081
Model 1	1.49		1.67		1.66		1.9	0.1679
Model 2	1.38^a^		1.69^b^		1.71^b^		6.2	0.0136

Means on the same row with the same superscript letter are not significantly different [*P* > 0.05].

Means on the same row as a potential confounding variable have been adjusted statistically for that variable.

For the meat exchange categories, low meat intake included women in the 1st quartile, daily servings per 4186 kJ [1000 kcal], 0.27–0.95, moderate meat exchange included women in the 2nd and 3rd quartiles, daily servings per 4186 kJ [1000 kcal], 0.96–1.59, and the high meat exchange category included women in the 4th quartile, daily servings per 4186 kJ [1000 kcal], 1.60–4.63.

Each of the exchange variables was calculated as servings per 4186 kJ [1000 kcal].

Model 1 includes the following covariates: age, education, body fat percentage, total physical activity, and servings of the following exchanges per 4186 kJ [1000 kcal], sweets, fruit, nonstarchy vegetables, dairy, very lean meat, and fat.

Model 2 employed the same covariates as Model 1, except that body fat percentage was not included.

**Table 3:** Mean differences in insulin resistance [HOMA] across the very lean meat [VLM] category, without and with means adjusted for the potential confounders.

Variable controlled	Very Lean Meat Exchange Group [*n* = 292]						*F*	*P*
	Low VLM intake		Moderate VLM intake		High VLM intake			
	[1st quartile]		[2nd-3rd quartiles]		[4th quartile]			
	[*n* = 73]		[*n* = 146]		[*n* = 73]			
	Mean	SD	Mean	SD	Mean	SD		
None	1.75	1.25	1.56	1.01	1.61	0.89	1.0	0.3284
Age	1.75		1.56		1.61		1.0	0.3223
Education	1.79		1.58		1.63		1.0	0.3087
Energy from carbohydrate	1.76		1.57		1.58		1.4	0.2362
Energy from protein	1.81		1.58		1.50		2.4	0.1261
Energy from fat	1.74		1.56		1.61		0.8	0.3755
Total energy intake	1.70		1.55		1.67		0.1	0.7571
Starch exchanges	1.76		1.55		1.61		1.1	0.2874
Sweets exchanges	1.78		1.55		1.59		1.5	0.2238
Fruit exchanges	1.75		1.56		1.60		1.1	0.3013
Vegetable exchanges	1.75		1.55		1.61		0.9	0.3359
Dairy exchanges	1.74		1.56		1.62		0.7	0.4036
Meat exchanges	1.75		1.56		1.60		1.0	0.3286
Fat exchanges	1.75		1.56		1.61		0.8	0.3815
BMI	1.76		1.57		1.57		1.7	0.1967
Body fat percentage	1.75		1.56		1.61		1.2	0.2830
Physical activity	1.75		1.56		1.60		1.0	0.3285
Model 1	1.73		1.55		1.64		0.5	0.4833
Model 2	1.71		1.55		1.62		0.4	0.5432

Means on the same row as a potential confounding variable have been adjusted statistically for that variable.

For the very lean meat [VLM] exchange categories, low VLM intake included women in the 1st quartile, daily servings per 4186 kJ [1000 kcal], 0.00–0.32, moderate VLM exchange included women in the 2nd and 3rd quartiles, daily servings per 4186 kJ [1000 kcal], 0.33–1.04, and the high VLM exchange category included women in the 4th quartile, daily servings per 4186 kJ [1000 kcal], 1.05–6.73.

Each of the exchange variables was calculated as servings per 4186 kJ [1000 kcal].

Model 1 includes the following covariates: age, education, body fat percentage, total PA, and servings of the following exchanges per 4186 kJ [1000 kcal], starch, sweets, fruit, nonstarchy vegetables, dairy, meat, and dietary fat.

Model 2 employed the same covariates as Model 1, except that body fat percentage was not included.

**Table 4:** Odds of insulin resistance [HOMA-IR ≥ 75th percentile] in women with low meat or low VLM intake compared to women with moderate to high intake.

Outcome: HOMA [≥75th percentile]	Meat intake per 4186 kJ [1000 kcal]		VLM intake per 4186 kJ [1000 kcal]	
	^*∗*^Low versus all others [*n* = 292]		^*∗*^Low versus all others [*n* = 292]	
Variable controlled	OR	95% CI	OR	95% CI
None	0.33	0.16–0.71	1.15	0.63–2.10
Age	0.33	0.15–0.70	1.15	0.63–2.09
Education	0.33	0.16–0.70	1.16	0.63–2.13
Percent of energy from carbohydrate	0.33	0.15–0.74	1.19	0.65–2.18
Percent of energy from protein	0.34	0.16–0.72	1.25	0.66–2.35
Percent of energy from fat	0.33	0.15–0.72	1.13	0.62–2.07
Total energy intake	0.32	0.15–0.70	0.95	0.50–1.78
Starch exchanges	0.30	0.14–0.65	1.18	0.65–2.16
Sweets exchanges	0.33	0.15–0.70	1.22	0.66–2.25
Fruit exchanges	0.34	0.16–0.74	1.16	0.63–2.11
Vegetable exchanges	0.33	0.16–0.71	1.15	0.63–2.10
Dairy exchanges	0.32	0.15–0.69	1.14	0.62–2.07
VLM exchanges	0.34	0.16–0.72	—	—
Meat exchanges	—	—	1.15	0.63–2.10
Fat exchanges	0.34	0.16–0.73	1.11	0.61–2.04
BMI	0.45	0.20–1.00	1.21	0.64–2.32
Body fat percentage	0.47	0.21–1.03	1.09	0.57–2.10
Physical activity	0.33	0.16–0.71	1.15	0.63–2.10
Full Model 1	0.38	0.16–0.90	0.99	0.47–2.10
Full Model 2	0.34	0.14–0.83	0.93	0.43–2.01

All dietary exchange variables were calculated as servings per 4186 kJ [1000 kcal].

OR: odds ratio; odds of having insulin resistance [HOMA ≥ 75th percentile].

95% CI = 95% confidence interval.

^*∗*^“Low” included women in the lowest or 1st quartile of consumption, whereas “all others” included women in the 2nd, 3rd, or 4th quartile of meat consumption or VLM intake [very lean meat], those with a moderate or high number of exchanges.

Odds ratios on the same line as a potential confounding variable were adjusted for differences in that covariate.

Full Model 1 included the following covariates: age, education, percent of energy from fat, percent of energy from protein, BMI, number of exchanges per 4186 kJ [1000 kcal] from starch, sweets, fruit, nonstarchy vegetables, dairy, fat, VLM [very lean meats], or meats [depending on the predictor], and objectively measured physical activity.

Full Model 2 included all of the covariates of Model 1 and also body fat percentage.
